# Undergraduate research in medical education: a descriptive study of students’ views

**DOI:** 10.1186/1472-6920-14-51

**Published:** 2014-03-17

**Authors:** Cristiano C Oliveira, Renata C de Souza, Érika H Sassaki Abe, Luís E Silva Móz, Lidia R de Carvalho, Maria AC Domingues

**Affiliations:** 1 Pathology Department, Botucatu School of Medicine, São Paulo State University, São Paulo 18618-000, Brazil; 2 Botucatu School of Medicine, São Paulo State University, São Paulo 18618-000, Brazil; 3 Biostatistics Department of the Botucatu Biosciences Institute, São Paulo State University, São Paulo 18618-000, Brazil

## Abstract

**Background:**

Medical students engage in curricular and extracurricular activities, including undergraduate research (UR). The advantages, difficulties and motivations for medical students pursuing research activities during their studies have rarely been addressed. In Brazil, some medical schools have included undergraduate research into their curriculum. The present study aimed to understand the reality of scientific practice among medical students at a well-established Brazilian medical school, analyzing this context from the students’ viewpoint.

**Methods:**

A cross-sectional survey based on a questionnaire applied to students from years one to six enrolled in an established Brazilian medical school that currently has no curricular UR program.

**Results:**

The questionnaire was answered by 415 students, 47.2% of whom were involved in research activities, with greater participation in UR in the second half of the course. Independent of student involvement in research activities, time constraints were cited as the main obstacle to participation. Among students not involved in UR, 91.1% said they favored its inclusion in the curriculum, since this would facilitate the development of such activity. This approach could signify an approximation between the axes of teaching and research. Among students who had completed at least one UR project, 87.7% said they would recommend the activity to students entering the course.

**Conclusion:**

Even without an undergraduate research program, students of this medical school report strong involvement in research activities, but discussion of the difficulties inherent in its practice is important to future developments.

## Background

Society holds important expectations of health professionals. Aside from their biomedical training, these include an active critical posture in relation to planning and conducting research aimed at increasing current knowledge, especially that which improves the living conditions and health of the general population [1,2].

In this relatively new professional context, universities from different countries are concerned about preparing medical students to meet the changing needs of society. The modern university is based on the triad of teaching, research and outreach activities and its application to medical training simultaneously requires technical and theoretical elements, the maturation of critical thought, development of the capacity for initiative, stimulation of independent self-directed learning and a sound approach to problem solving, whether basic or clinical science [3-8].

Undergraduate research (UR) has become an integral part of medical education in numerous countries and has influenced the subsequent performance of physicians, yielding positive results in the development of important skills, including critical analysis and leadership, whether or not the professional pursues an academic or research career [3-5,7,9].

According to medical students, UR is motivated mainly by a desire to improve learning, while endeavoring to increase selection chances in residency or specialization exams [4,6-10], as such, universities should offer opportunities for students to participate in the continued advancement of knowledge.

Recognizing the relevance of this type of extracurricular activity, numerous Brazilian universities have included it in discussions concerning the curriculum of medical courses, while in some, undergraduate research is integrated into the course [8,9]. The literature presents some experiences of linking disciplinary curricula for UR with the first year of medical school that quantitatively evaluate mandatory or elective programs [8,11], focusing on their production [9], citation impact [4,11], teacher/student involvement [4,7], number of projects developed and distribution of these in diverse medical areas [8,9]. However, the advantages, difficulties and motivations for medical students pursuing research activities during their studies have rarely been addressed [3,4,9].

The present work aimed to understand the reality of scientific practice among medical students at a well-established Brazilian medical school, determining factors that drive or hinder the pursuit of undergraduate research and analyzing the context of scientific practice during the undergraduate course from the students’ viewpoint.

## Methods

The study design consisted of a cross-sectional model applied to 540 medical undergraduate students from years one to six, enrolled in the academic year of 2009 at Botucatu School of Medicine (*Faculdade de Medicina de Botucatu*, FMB) of São Paulo State University (UNESP), irrespective of their involvement in scientific research.

The FMB-UNESP is a Brazilian public institution that was founded in 1962, in which education, health care and research are interlinked. Its medical curriculum is structured in a traditional model: in the first two years, the students are involved in disciplines of basic health sciences; during the years three and four, they initiate the applied phase of the course; and final two years focus on internship. Medical students attend theoretical classes and participate in practical activities at all levels of health care. The students also develop extracurricular activities, including outreach, social, sports and research activities (undergraduate research).

No structured curricular program for research exists within the undergraduate course at the FMB. Thus, medical students who wish to conduct research activities during their undergraduate course need to find professors that are willing and available to orient their projects.

The study was approved by the Board of Undergraduate Medicine and the Research Ethics Committee of the FMB-UNESP. Students were invited to participate in the study and a term of free informed consent was signed prior to participation.

A semi-structured questionnaire, including questions and open-ended comments, was designed using input from undergraduate students, following a pilot study (see Additional file 1). The questionnaire divided the students into those involved in or who had participated in UR, and those not involved or who did not intend to participate. The latter group answered six questions concerning general principles: main contribution to student formation; main obstacle to participation; information provided by teachers; the value of a Scientific Method discipline and whether it would facilitate access to UR; and whether fixed periods in the curriculum would favor participation. The former answered 12 specific questions: reasons for pursing UR; main obstacles to participation; reasons for choosing the department and supervisor; whether UR increased their interest in the subject; possible contributions of UR to student learning; whether the supervisor organized meetings; their main expectation upon project completion; the importance of grades, extension activities and monitoring; whether they received a grant; and what course year they initiated their project. A subgroup of those who had completed at least one project answered five additional questions: how many completed projects; their duration; whether the results were published; whether the project influenced their decision regarding specialization; and whether they would recommend UR to students of the first year of medical course.

Descriptive analysis was performed on all quantitative variables and expressed as percentages. Some references to student comments are included for clarification.

## Results

### General data

The questionnaire was answered by 415 students (yielding a response rate of 76.8%) from years one to six of the undergraduate course; the majority were women (n = 260, 62.7%). Among these 415 students, 18.8% were from first year, 18.3% were from second year, 18.8% were from third year, 15.18% were from fourth year, 16.62% were from fifth year and 12.3% were from sixth year.

The survey revealed that 219 (52.8%) students had not participated in any research project during undergraduate education. And 196 (47.2%) medical students had participated in some type of research project during undergraduate education.

### Undergraduate research and motivation

The survey revealed that of the 219 (52.8%) students who had not participated in any research project during undergraduate education, 187 (85.4%) showed an interest. This group felt that the greatest contributions of UR to their training were increased medical knowledge (43.4%) and curriculum enrichment (29.2%), while 32 reported no plans for such activity.

**Figure 1:**
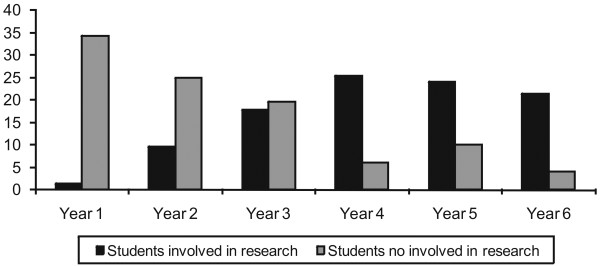
Percentage of FMB-UNESP undergraduate students involved in UR according to course year.

The percentage of students involved in UR exceeded those not involved from year four onward (Figure 1). Development of a student’s first project began during year two of the course for 40.3%, followed by year three (36.2%), year four (13.3%), first year (7.1%) and during internship (2.0% in year five, 0.6% in year six). Among undergraduate students involved in research, 100 (51.0%) were conducting an UR project in June 2009, 25 (12.8%) were involved in more than one project in different departments and 71 (36.2%) had completed at least one UR project during the course.

Analysis of these data also revealed that student motivation to participate in UR (Table 1) was related to curriculum enrichment (32.1%), associated with the need for a grant (19.9%) or the chance to increase their expertise in a particular area (17.3%). Grouping only fourth to sixth-year students, curriculum enrichment was cited by 80.1%. A small group (1.0%) mentioned other reasons for pursuing UR, including understanding the scientific method and learning how to design research projects.

**Table 1:** Motivation behind the pursuit of UR among FMB-UNESP undergraduate students involved in research

	**Percentage**
Curriculum enrichment	32.1
Curriculum enrichment + The need for a grant	19.9
Curriculum enrichment + Improving expertise in a particular area	17.3
Improving expertise in a particular area	10.2
Curriculum enrichment + Research forms part of their plans as future professionals	7.7
Improving expertise in a particular area + The need for a grant	3.1
Research forms part of their plans as future professionals	2.0
Curriculum enrichment + Other reasons	2.0
Research forms part of their plans as future professionals + Improving expertise in a particular area	2.0
The need for a grant	1.0
Other reasons	1.0
Research forms part of their plans as future professionals + The need for a grant	1.0
Improving expertise in a particular area + Other reasons	0.7
	100

Concerning curriculum enrichment, the attributes deemed most important were the grades obtained during undergraduate studies (52.0%), followed by extension activities, such as UR (38.6%), and monitorships in specific areas (9.6%). Reservations concerning the consistency of grades, their reflection on learning and their influence in residency exams and project submissions to funding agencies were discussed in students’ comments.

Regarding financial support from agencies that promote research development, 47.4% of the students received UR grants, 23.7% from the São Paulo Research Foundation (FAPESP) and 23.7% from the National Council for Scientific and Technological Development (CNPq) (data provided by the Institutional Grant Program for Undergraduate Research, PIBIC). We should add that 22.4% of respondents received no financial support while participating in UR.

The study’s objectives influenced the choice of the department in which 24.5% of respondents sought participation in UR, while the classes of the discipline influenced this choice for 19.4%, a very similar percentage to those who indicated that they intended to follow a specialization in the same department. The influence of positive experiences with colleagues in the department chosen was cited by 16.8% of respondents, while 8.2% cited the department’s tradition as justification. Associations between these topics were cited by 11.2%.

### Undergraduate research and difficulties

Considering the respondents not involved with research, the two most important difficulties in implementing an UR project were the availability of time (67.7%) and finding a professor willing to orient and develop the research (21.2%) (Table 2). In agreement with these data, 64.1% of the respondents indicated that aggregating free periods to their timetables was fundamental for conducting scientific research; however, 32.0% recognized the importance of such periods, but claimed their absence was not the main obstacle. Respondents commented that additional time to conduct this type of activity would influence the quality of the projects developed, would enable clearer definition in the planning, organization and implementation of the same and assist in contacting a research supervisor (see Additional file 2).

**Table 2:** The principal difficulties in conducting UR at FMB-UNESP according to the students

	**Percentage**
Availability of time	67.7
Problems involved in finding a supervisor willing to orient and develop the research	21.2
Projects completed but not presented at congress or published in journals.	10.4
Other reasons	0.7
	100

In addition, 180 (82.2%) students from this group affirmed the lack of information and dissemination of UR within the institution as a difficulty in implementing research, particularly in the first two years. Unfamiliarity with the concept, its importance, implementation, specific projects and the lack of contact with supervisors were all discussed.

The difficulties cited by non-participating students showed similarities with those involved in UR. For 50.2%, the availability of time was the principal obstacle, followed by problems concerning supervisor collaboration (14.2%) and lack of research funding (0.9%). Comments indicated not only the lack of time available among students, but also among professors/research supervisors.

### The relationship between medical student and the research supervisor

FMB-UNESP students who want to conduct research need to find a relevant opportunity with a teacher. Among students involved in research, professors with time available to develop the project were the most frequently chosen, according to 32.1% of respondents. Other important points in this choice included the teacher’s motivation to conduct research (16.8%) and the didactic demonstrated in undergraduate classes (11.7%). A combination of these factors was reported by 5.1%. For 15.8%, colleagues who had developed projects with a particular supervisor were an important influence. Other reasons discussed included the influence of academic leagues/tutorials, the teacher’s capacity and curriculum, direct invitation and interest in the teacher’s line of research.

Students assessed supervisor involvement in organizing group meetings to discuss research projects and methodologies and promote research team integration. According to 49.0% of respondents, such meetings were organized and for 41.9%, they proved useful and were considered necessary to initiate productive activities, while 7.1% stated they were not very objective. Most respondents (51.0%) confirmed that no meetings occurred, but the proposal was considered interesting by 84.3% of this subgroup.

### Undergraduate research and curriculum

Among non-participating students, 91.3% affirmed curricular inclusion would facilitate the pursuit of UR, because the scientific theory would be better understood and would enable greater contact with the teachers. However, even though a specific discipline for scientific practice was considered important (91.1%), 74.9% indicated it should be elective. For 1.2%, others advantages included providing space for UR and improving the quality of the projects developed by students. In contrast, 7.2% of students alleged that a specific discipline would not facilitate UR, rather research should be spontaneous, not an obligation.

### Undergraduate research: contributions and expectation

Positive contributions of UR to learning were perceived by 63.8% of respondents during graduation. Another 33.2% affirmed they felt no specific contribution, though 16.8% expected to perceive some impact of UR in their future careers. For 91.8% of respondents, UR stimulated increased interest in the subject under investigation, with 33.2% recognizing its importance and 59.2% reporting improved understanding of the subject. It is worth highlighting that 8.2% of respondents confirmed no enhanced interest in the subject and 3.1% of the group believed UR contributed nothing to learning, concluding that its merits were restricted to curriculum enrichment.

The principal expectation cited by 65.3% of respondents concerning their projects was the publication/presentation of their findings at conferences, followed by learning scientific methodology and understanding the subject, 10.2% each. Developing critical understanding of medical publications was cited by 10.7%, while a combination of these factors was reported by 3.6% of students. Some students (28.0%) claimed interest in a university career, with 4.1% expressing a desire specifically related to the FMB-UNESP. Curiously, despite strong involvement in UR, only 2.0% of participating students confirmed clear aspirations of becoming researchers.

Students who had completed at least one UR project answered five additional questions. Among these, 67.9%, 21.9% and 10.2% had already completed one, two and three or more UR projects, respectively.

The majority of completed projects (67.9%) were presented at scientific meetings/congresses, 42.3% with a good chance of publication in an indexed journal and 25.6% that were unlikely to be published. Congress presentation plus article publication was achieved by 16.7% of the projects, while 5.1% were only published and 10.3% of the projects completed have yet to publicize their results (Table 3).

**Table 3:** Final result of the projects developed by FMB-UNESP undergraduate students

	**Percentage**
Presented in congress, but not yet published in a journal.	42.3
Presented in congress, but will not be published.	25.6
Presented in congress and published in a journal.	16.7
Project completed but not yet presented at congress or published.	10.3
Published in a journal.	5.1
	100

Students who had terminated a UR project were asked whether they would recommend participation in research activities to those beginning medical school. The majority (88.7%) confirmed they would recommend such activities, while clarifying that UR produces more positive effects from year two or three onward.

## Discussion

The percentage of students involved in academic research in FMB-UNESP was higher than in a similar Brazilian study (28%) [12], but lower than in Norway (87%) [13]. According to several authors, the reasons that prevent students from participating in research activities range from lack of student awareness, to physical infrastructure deficits and unmotivated university staff, with some emphasis on ineffective institutional incentives to conduct UR [3,12]. The issue of stimulus for UR was raised by the students, who discussed several essential factors: information regarding the concept, its importance, contact with research supervisors, project execution and the provision of adequate information by teachers.

Analysis verified that the number of students involved in research increased from the first year, superseding those not involved from year four onward, when involvement peaked. European studies confirm that year two or three is the most likely period of UR initiation [4,13]. As the undergraduate course advances, a better foundation exists for students to conduct research in different fields of medicine, including showing concern for curriculum enrichment due to the appreciation of UR in interviews and for grant requests, until the students become involved in internship. During internship, the students concentrate on developing professional skills and preparing for medical residency exams [14].

Regardless of their involvement in UR projects or not, FMB-UNESP students reported lack of time as the primary obstacle to research. The rates reported here are substantially higher than in other Brazilian studies (23.7% [9], 10.1% [15]), differences that are probably related to the type of research and the course structure [9]. The FMB-UNESP teaching curriculum provides no fixed free periods, making it difficult to organize time for research projects and contacting supervisors, who also have rigorous schedules for teaching and assistance activities. For 63.9% of non-participating students, fixed free periods within the curriculum timetable are fundamental to student involvement in research. This issue appears more resolved in certain developed countries, particularly the USA [16].

Relationships with supervisors were cited as a difficulty by all students, primarily due to non-collaborative supervisors. Sarinho et al [9] reported that 9.6% of students mentioned this issue [9]; however, similar reports were not identified in European studies [4,13]. Discontinuity or difficulties in managing UR projects is linked to student demotivation, primarily centered on poorly integrated relationships with supervisors [17]. In this dynamic, the supervisor should have greater knowledge and thus their handling of this position influences the student/teacher relationship; the form of language used, their ability to express themselves, their skills set, posture and attitudes when conducting meetings and managing adverse factors are closely related to the success of supervision [18,19]. In agreement with these factors, the choice of supervisor by FMB-UNESP students considering UR was associated with teacher accessibility and influenced by the question of availability. Following these, the students discussed factors related to the teacher’s personal characteristics, including encouraging student involvement and didactic approach during class. Good performance in educational activities by a particular department was also important in this process, as were experiments successfully completed by the department and its teachers in previous research activities.

At the FMB-UNESP, UR students affirmed that the main reason they became involved was curriculum enrichment, similar to that observed elsewhere in Brazil [9,15] and other countries [4-7,13,16]. More than 50% of UR students were in the second half of the medical course, coinciding with the period when students focus on improving their curricula for employment and residency exams and interviews. Among non-participating students, the main contribution of UR to professional training was knowledge acquisition, followed by curriculum enrichment. The fact that most of those not involved in UR (84.0%) were in the first half of the course, when concerns about jobs and specialization are less prevalent, likely explains this difference.

In this study, only 47.2% of respondents confirmed they received UR grants, partially justifying why this issue is not an important motivator for participation, or a significant obstacle. Research grants are a valuable tool for the university and provide a social component to UR by collaborating in student maintenance, allowing them to invest in their studies [15]. Similar considerations were not identified in other studies, suggesting that in developing countries, a financial motive exists for pursing UR that does not influence medical students in developed nations, like the USA, Canada and Europe.

Promotion of regular group meetings to discuss ongoing projects was positively evaluated by the respondents (49%). Such meetings were also considered interesting by students involved in research which lacked this component during the execution of a project. Informative meetings and training sessions within the context of a research group are valuable, since they allow students contact with other research methodologies and subjects and permit interaction with other researchers.

Regarding the expectations of the students involved in UR, the main ones were presenting results at scientific meetings and journal publications, affirmed by 65.3% of the respondents. Students of an elective course in Canada (Critical Enquiry), held similar expectations (47% presentation, 76% publication) regarding future involvement in research [11]. Our analysis verified that among those who completed at least one project, 67.9% presented the results at scientific congresses and 16.7% achieved publication. This rate is favorable compared with another Brazilian study [9], where 81.5% of the work was neither published nor presented at scientific congresses, and is comparable to that verified for Dutch medical students, where 14,5% of the medical students published at least one scientific paper during the last three years of the medical course [5]; however, the quality and impact of FMB-UNESP student publications was not assessed here. Considering published articles involving student authors, the total volume for the FMB-UNESP is still lower than research intensive programs at Stanford University School of Medicine, where 90% of students were involved in research and 75% of undergraduates had published an article as the primary author as early as 1995 [16]. The longest running UR course in Brazil dates from 1995, but the results of this activity on medical education are far less consolidated than those reported by Stanford [14,16].

Similar to UR worldwide FMB-UNESP students evaluated UR positively, in that 63.8% of the respondents perceived the contributions of UR to their education at undergraduate level, a finding reinforced by their increased interest in the subject studied. Corroboration that UR is a positive experience is provided by the high percentage of students who would recommend UR to first-year medical students (88.7%). Nevertheless, students qualified their observations regarding the timing of such projects, recommending year two or three of the course, coinciding with the fact that 76.5% of them also initiated their projects at this stage of undergraduate education. Correspondingly, UR was evaluated positively at Stanford University, with 79% of the students expressing satisfaction, while affirming they were motivated to consider research (75%) and academic careers (60%) [17]. Among students involved in UR at the FMB-UNESP, 28.0% were considering academic careers; however, comparisons should consider the cultural, socioeconomic and temporal differences between the various studies available.

UR contributes to developing medical professionals with the ability to integrate scientific methodology and reasoning into their clinical practice and who pursue continuous improvement and upgrading [20-23]. Individuals who participate in research activities during undergraduate education, including future non-researchers, develop leadership skills that enable local/regional actuation in the context of their profession and specialization [14,17,22-24]. Recent studies have affirmed that UR students show improved communication skills, develop critical analysis and are successful in selection programs for postgraduate studies/medical residencies and in their working lives, achieving academic and/or professional titles faster, while presenting distinguished accomplishments in their professionalism and capacity [22,23,25].

That this context shows such favorable aspects for UR further provokes the discussion concerning its inclusion in medical school curricula, in Brazil or elsewhere. In our study, 90.9% of students not involved in UR believe a discipline focused on scientific methodology within the undergraduate course is important and would facilitate access to UR.

Research within the curricula of medical schools is part of a recent trend in medical education. Medical education is currently diversifying its scenarios to include emergency medicine and primary health care, following educational models that are also centered on the students as generators of knowledge. Studies suggest that research is an essential element in the formation of the new health professional [19,22,23,25], and ways to promote its inclusion are being discussed on campus and in the literature [26].

Proponents of the elective form believe that the time devoted to such training could be directed to other curricular activities, arguing that research data analysis is a task better performed by expert advisers appointed to preselect such contents. Those in favor of mandatory disciplines believe that the benefits of UR extend well beyond the limits of interpreting literature articles, providing an entire skills set that students can acquire in the development, implementation and dissemination of scientific work [4,9,20,22], redefining this activity as a tool of medical education to construct a new profile of the health professional. Recent Brazilian legislation aims to promote integration among medical education, the health system and society’s needs, capable of producing healthcare that is relevant to the community [27]; considering the benefits of undergraduate research in enabling future doctors, this should be considered an essential element in the continuing development of the medical curriculum in developing countries.

## Conclusions

This research aimed to characterize undergraduate research in Brazilian medical school with a classic curriculum model. The survey was structured in a questionnaire of simple answers with space for open-ended comments. Despite these aspects, the study sought to understand student perception of UR in the institution studied in order to encourage reflection regarding new trends in the local medical curriculum.

At the FMB-UNESP, an important part of the students is involved in UR, this activity was well evaluated by students and the destination for the majority of projects is presentation of the results at scientific congresses. However, within the institution, there is no curricular program or similar structural incentive for UR. According to students not yet involved in research activities, implementing this type of discipline, whether mandatory or elective, could facilitate access to UR and minimize obstacles regarding the availability of time, making contact with supervisors, disseminating projects/lines of research and understanding scientific methodology, while elucidating the importance of UR and it usefulness in the practice of health professionals. Moreover, this approach could signify greater approximation between the axes of teaching and research, attracting more teacher-researchers to undergraduate education.

Undergraduate research at FMB-UNESP was characterized from the students’ viewpoint, providing important insights that could prove relevant to curriculum development. The medical students of the FMB-UNESP recognize the importance of UR in relation to their professional training and in understanding the influences of scientific practice in undergraduate medical education.

## Competing interests

The authors each individually and collectively declare there are no conflicts of interest.

## Authors’ contributions

The authors COO, RCS, EHSA and LESM participated in the design of the project, conducted the literature review, participated in the design of questionnaires, conducted the field work and contributions awarded to the final version of the article. The author LRC was responsible for the statistical analysis and participated in data interpretation. The author MACD was the principal investigator for the project. All authors approved the final version of the article.

## Pre-publication history

The pre-publication history for this paper can be accessed here:

http://www.biomedcentral.com/1472-6920/14/51/prepub

## Supplementary Material

Additional file 1 Questionnaire administered to students of medicine of Botucatu School of Medicine. Click here for file

Additional file 2 A selection of comments made by the students in the open-ended questions and spaces for comments. Click here for file
