# Supplementary material for: Undergraduate research in medical education: a descriptive study of students’ views
Source: BMC Med Educ. 2014 Mar 17;14:51. doi: 10.1186/1472-6920-14-51 (PMC4021277; doi:10.1186/1472-6920-14-51)
Supplement: Additional file 1 — Questionnaire administered to students of medicine of Botucatu School of Medicine. [file 1472-6920-14-51-S1.doc]

**Additional file 1:** Questionnaire administered to students of medicine of Botucatu School of Medicine.

We would like to clarify that the translation of this questionnaire has tried to anticipate certain cultural differences between Brazil and English-speaking countries regarding eliciting student responses.

**Questionnaire** – Project “Factors influencing the pursuit of undergraduate research (UR) at the Botucatu Medical School (FMB/UNESP) and its consequences among undergraduate students”

| Age: | Sex: ( ) FEMALE ( ) MALE |
| --- | --- |
| Year: ( ) 1^st^; ( ) 2^nd^; ( ) 3^rd^; ( ) 4^th^; ( ) 5^th^; ( ) 6^th^. | |
| Do you participate in the “PET[[1]](#footnote-2)” program? ( ) Yes ( ) No | |

1. Not including the “PET” program, in relation to UNDERGRADUATE RESEARCH (UR):

A) You are participating in an UR project.

B) You are participating in more than one UR project in different departments.

C) You are not participating in an UR project, but intend to.

D) You are not participating in an UR project and do not intend to.

E) You have already concluded at least one UR project during the undergraduate course.

**If you responded options C or D, please answer questions numbered 1 to 7, entitled General Questionnaire.**

**If you responded options A, B or E, please answer questions numbered 1 to 16, entitled Specific Questionnaire.**

**General Questionnaire: for students not participating in Undergraduate Research (UR)**

1. What would be the main contribution of UR to your education?

A) Acquiring knowledge.

B) An opportunity to contribute to new discoveries.

C) Learning scientific methods.

D) Improving your Curriculum Vitae.

E) Other reasons. Please specify: ____________________________________________________

______________________________________________________________________________

2. What is the main difficulty in participating in UR at the FMB?

A) Availability of time (lack of free periods).

B) Lack of cooperation on the part of researchers.

C) Difficulty finding a supervisor who is willing to teach.

D) Lack of funding.

E) Other reasons. Please specify: __________________________________________________

________________________________________________________________________________

3. In the classroom, do the teachers talk about UR, its importance and its possibilities?

A) Yes, a minority.

B) Yes, the majority.

C) No.

4. Do you believe it is important to add a “Scientific Methodology” discipline to the undergraduate medical course?

A) Yes, as a compulsory discipline.

B) Yes, as an elective discipline.

C) No.

Please comment: ___________________________________________________________

_____________________________________________________________________________

_______________________________________________________________________________

5. Do you believe that a “Scientific Methodology” discipline would facilitate the search for UR?

A) Yes, because it would be easier to find a supervisor.

B) Yes, because I would have a better understanding of scientific theory as well as its importance and usefulness.

C) Yes, for the reasons stated in A and B.

D) Yes, for another reason. Please clarify: ______________________________________________

E) No.

Please comment: ___________________________________________________________

_____________________________________________________________________________________________________________________________________________________________

6. Do you believe that the adding fixed free periods to the timetable would favor participation in UR by medical undergraduates of the FMB/UNESP?

A) Yes, free periods are critical to the execution of UR.

B) Yes, free periods are important, but the lack of them is not the main obstacle.

C) No, the lack of free periods does not represent an obstacle to participation in UR.

7. Space for general comments: ______________________________________________________

______________________________________________________________________________

_____________________________________________________________________________

Specific Questionnaire: for students who are participating in or have completed an Undergraduate Research (UR) project

1. Why are you participating or have you participated in undergraduate research (UR) activities? Check a maximum of two options.

A) To improve your curriculum.

B) Being a researcher forms part of your plans for the future.

C) The potential to improve your knowledge in a specific area.

D) You need or needed a UR grant.

E) Other reasons. Please specify: __________________________________________________

________________________________________________________________________________

2. What are the main difficulties in conducting UR at the FMB/UNESP? Check a maximum of two options.

A) The availability of time.

B) The lack of cooperation from researchers.

C) The difficulty of finding a supervisor who is willing to teach.

D) Lack of funding.

E) Other reasons. Please specify: __________________________________________________

________________________________________________________________________________

3. Why did you choose the department(s) concerned?

A) The department has a tradition.

B) The undergraduate classes sparked an interest in the research developed by the department.

C) I intend to follow a speciality in the department.

D) Colleagues recommended the department to me.

E) The objectives of the research seemed interesting.

4. What is the main reason that led you to choose the supervisor in question?

A) The supervisor was accessible.

B) The classroom teaching the supervisor demonstrated.

C) The supervisor motivated me to do research.

D) Colleagues informed me that received support from the supervisor in previous studies.

E) Because I had no other option.

F) Other reasons. Please specify: __________________________________________________

________________________________________________________________________________

5. Has UR increased your interest in the subject you researched?

A) Yes, I now recognize its importance.

B) Yes, now I understand the subject better.

C) No.

6. Regarding the possible contributions (income, oral presentation and article publication) of UR on your learning:

A) You perceive or have perceived them during the undergraduate course.

B) You have no perception of them during the undergraduate course.

C) You have no perception of them yet, but expect to during your professional career.

D) It made no contribution; UR is restricted to improving your curriculum.

7. Does/Did your supervisor hold research group meetings?

A) Yes, and these meetings are necessary to initiate productive activities.

B) Yes, but they are dull and not very objective.

C) No, but I think they would be an interesting proposition.

D) No, I do not consider them important during UR.

8. What is or was your main expectation after completing the project?

A) The publication of the results and/or presentation at conferences.

B) Learning scientific methodology.

C) An in-depth knowledge of the subject under study/studied.

D) The development of critical reasoning in relation to papers published in the medical field.

9. Are you interested in an academic career?

A) Yes, but only at UNESP.

B) Yes, in some university.

C) Only as a second option.

D) No.

E) I don’t know.

10 List the following tasks in order of importance for enriching your curriculum (1^st^, 2^nd^, 3^rd^).

( ) Take good notes graduation.

( ) Extension activities (e.g. UR, activities in the academic center, in sports, in academic leagues).

( ) Tutoring in specific areas.

Please comment: ________________________________________________________________

______________________________________________________________________________

_____________________________________________________________________________

11. During the project, do/did you receive support in the form of a grant?

A) Yes, from the FAPESP[[2]](#footnote-3).

B) Yes, from the PIBIC/CNPq[[3]](#footnote-4).

C) No.

D) I do/did not receive an UR grant, but I do/did receive a grant for some other activity, such as monitoring, CEATOX, PAE, PROEX[[4]](#footnote-5).

E) No, the research project is still under review.

12. In what year you initiate your first UR project?

A) 1^st^ year

B) 2^nd^ year

C) 3^rd^ year

D) 4^th^ year

E) 5^th^ year

F) 6^th^ year

**Questions 13 through 17 should be answered only by those who have completed at least one undergraduate research (UR) project.**

13. How many UR projects have you completed?

A) One.

B) Two.

C) Three or more.

14. How long did this(these) project(s) take? _­­­__________________________________________

____________________________________­­_________________________________________

15. The end result of your project was:

A) It was published in a scientific journal.

B) It was presented at congresses, and will eventually be published.

C) It was presented at congresses, but will not be published.

D) It was presented at congresses and published in a scientific journal.

E) It was completed, but has not yet been presented at a congress or published in a journal.

16. The development of this project influenced your decision regarding your choice of residency speciality?

A) I haven’t chosen my speciality yet.

B) Yes, my area of choice is the same as the UR project.

C) No, my area of choice is different from the UR project.

17. Would you recommend participating in UR to someone who is now entering medical school?

A) Yes.

B) No.

Please comment: ________________________________________________________________

______________________________________________________________________________

______________________________________________________________________________

1. ### PET, Working in Health Care Education Program (*Programa de Educação pelo Trabalho para a Saúde*)

   [↑](#footnote-ref-2)
2. FAPESP, São Paulo Research Foundation. [↑](#footnote-ref-3)
3. PIBIC/ CNPq, Institutional Program of Scientific Initiation (UR) Grants / National Counsel of Technological and Scientific Development [↑](#footnote-ref-4)
4. CEATOX, Toxicological Assistance Center (FMB); PAE, Institutional Student Support (UNESP); PROEX, Academic Support and Extension (UNESP) [↑](#footnote-ref-5)
