# Supplementary material for: Undergraduate research in medical education: a descriptive study of students’ views
Source: BMC Med Educ. 2014 Mar 17;14:51. doi: 10.1186/1472-6920-14-51 (PMC4021277; doi:10.1186/1472-6920-14-51)
Supplement: Additional file 2 — A selection of comments made by the students in the open-ended questions and spaces for comments. [file 1472-6920-14-51-S2.doc]

**Additional file 2: A selection of comments made by the students in the open-ended questions and spaces for comments**

**Concerning the availability of time for UR:**

*“The existence of a specific period for UR would allow for planning and organization of the work, with greater dedication.”*

*“Certain experiments require specific intervals for their evolution and in most cases, it’s impossible.”*

*“Fixed free periods would be very useful for us to organize ourselves in terms of time, studies and UR.”*

*“When the student gets some free time, they cannot reconcile it with that of the supervisor.”*

**Concerning the lack of information about UR:**

*“There’s a lack of information. I don’t know how to set up UR, or even what UR is.”*

*"There’s a lack of information and dissemination and students don’t know the contribution UR can make to academic and professional life.”*

*“I don’t know where to get information or how to join a project.”*

**Concerning a possible course on UR, students in favor:**

*“Provided that it was of quality and had clear objectives.”*

*“Provided that the teaching method was very interesting”*

*“Provided that it was delivered on time, not in the first year, and with specific objectives.”*

*“You would need enough time to study it as an elective.”*

**Concerning a possible course on UR, students not in favor:**

*“It would become one more marginalized subject that wouldn’t arouse the slightest interest in students.”*

*“UR should be sought out because you’re interested and not because it’s a discipline.”*

*“People who are interested will go for it without the need for a new discipline.”*

**Reasons for pursuing UR:**

*“Understanding and practicing the scientific method.”*

*“Learning how to do scientific work.”*

**Grades and their relation to UR:**

*“Even though I don’t consider that grades reflect learning.”*

*“Actually, I believe that extension activities are as important as good grades. However, in order to get grants in UR projects, we have to rely on a good record of grades in undergraduate studies, which is why I put them first.”*

*“The grades are important to get a grant for UR in some departments, and even a monitorship.”*

**The pros and cons of dedicating time to UR:**

*“It is worth emphasizing the importance given to publications in scientific journals and conducting studies in the department of future interest.”*

*“I believe the greater weight given to the extension activities diverts students from curriculum activities, which I believe are most important. In addition, teachers only value research, this ‘self-interest’ ends up harming a lot of graduation activities, such that are students often a secondary concern.”*

*“Faculties do not provide the basis for the complete formation of the student as a human being, who will have major responsibilities in the future, and there is no incentive to try to improve this*

**When UR should be included in the course:**

*“From the second or third year onward, due to greater maturity.”*

*“From the second year, I think before that, students do not have the knowledge necessary to develop this activity”*

**Reasons for recommending UR to younger students:**

*“It helped me realize that I like scientific research and today I look at scientific studies in a different way.”*

*“It’s definitely is very important for the professional growth of medical students”*

*“To obtain experience, maturity, knowledge, responsibility, critical analysis.”*

*“Because besides being a differential in your curriculum, students are encouraged to develop the methodologies necessary to write a paper and principally to perform oral presentation and design posters.”*

**Developing awareness of UR:**

*“UR should be more widely announced and edicts should be well publicized in the departments, because the students have to go looking for supervisors who are interested and are not always well received.”*

*“Nobody told me it was so important, I started too late”*
